# Enhancing healthcare leadership in Gujarat, India: an effectiveness study

**DOI:** 10.3389/fpubh.2025.1677824

**Published:** 2025-10-20

**Authors:** Jallavi Panchamia, Neha Chavda, Bharati Sharma, A. M. Kadri, Veena Iyer

**Affiliations:** ^1^Indian Institute of Public Health, Gandhinagar, Gujarat, India; ^2^State Health System Resource Center, Government of Gujarat, Gandhinagar, Gujarat, India

**Keywords:** healthcare, leadership program, effectiveness, Kirkpatrick framework, evaluation

## Abstract

**Background:**

The state government of Gujarat, India, recognized the need for a systematic leadership enhancement program in 2022 for mid- and senior-level government technical health officials to address complex health system challenges. The Health Leadership Enhancement Program (HLEP) was designed and implemented using the LEADS framework for approximately 150 Public Health and Healthcare leaders in Gujarat, in three cycles, to meet this demand.

**Methods:**

This paper aims to assess the effectiveness of the Healthcare Leadership Enhancement Program using Kirkpatrick’s four-level framework. We evaluated the program’s effectiveness through the four levels: Reaction, Learning, Behavior, and Results.

**Results:**

Over 97% of participants reported having significantly positive reactions to the program’s content and its relevance to their leadership roles. Most participants reported the highest learning under self-awareness and people management. The participants’ subordinates perceived a behavioral shift in the leadership approaches of their leaders. System-level changes were at the level of local work sites rather than at the broad policy level. The participants rated case studies, mentoring, and practice-based assignments as favorable methods under program pedagogy.

**Conclusion:**

Leadership enhancement programs designed to match work contexts and experiential pedagogy have the potential to enhance individual self-awareness and team management. Real-world case studies, mentoring, and practice-based assignments accompanied by classroom learning seem to be the better pedagogy.

## Background and rationale

There is a striking mismatch between the rising global demand for competent healthcare workers and the minimal investment in their training—just 2% of total health expenditures (1). In the context of LMICs most public health and healthcare leaders come from clinical and medical backgrounds and have little or no formal management training to exercise leadership roles (2, 3). Despite several years of experience, clinical training alone does not prepare medical professionals for management/leadership responsibilities (4, 5). New leaders find the responsibilities of effectively managing scarce resources, staff, funds, drugs, equipment, infrastructure, and collaborating with other sectors, challenging (6). There is a critical need for systematically enhancing leadership capacity to ensure effective delivery of public health and healthcare services. Formal leadership training within the public health sector is not institutionalized, leaving health professionals unprepared when transitioning into senior positions within state health departments (7, 8). Limited studies in health care organizations (9, 54) focus on capacity-building programs for the government’s public health and healthcare employees.

Gujarat States’ contribution to the national Gross Domestic Product (GDP) is 8.6%, which is higher than most Indian states. Gujarat is renowned for its business leadership, driven by an entrepreneurial spirit, innovation, efficiency, and data-driven decision-making (10). Such performance is lacking in Gujarat’s health system. Despite Gujarat’s success in reducing maternal and neonatal mortality, the state still needs to improve other healthcare outcomes, such as the prevalence of malnutrition and non-communicable diseases. Among children below 5 years of age, Gujarat ranks 2nd for the prevalence of underweight (39.7%) and 4th for stunting (39%), compared to other Indian states (11, 12, 52). Districts with predominantly tribal populations have a higher prevalence of malnutrition compared to the state’s average (50, 51). Although 82.9% of households had PMJAY (53) cards, only 43.3% used them, and 22.9% still incurred out-of-pocket expenses, as found in a study in Gujarat (13, 49).

As national and state-level investments in health increase, leading to the roll-out of many more prevention, control, and digital programs on the ground, the imperative to manage, lead, and collaborate among numerous heterogeneous groups of workers and departments has grown louder (14). Recognizing these challenges, the Government of Gujarat views enhancing the leadership competencies of health sector leaders as one key intervention to improve health system performance. The Indian Institute of Public Health Gandhinagar (IIPHG) in collaboration with the State Health Systems Resource Center (SHSRC) designed and implemented a Health Leadership Enhancement Program (HLEP) for mid-career health leaders working in the state’s health system since 2022. The program’s content was guided by the LEADS framework proposed by the Leadership Competencies for Public Health Practice in Canada (15).

Through this paper, we aim to document the components of the recently implemented HLEP and its pedagogical framework, as well as discuss the findings from an ongoing assessment of six initial batches of training of 148 mid-level public health and health care leaders of Gujarat. The findings from this assessment will feed back into the HLEP design and also serve as a valuable resource for the design and implementation of similar capacity- building efforts for strengthening leadership among public health and healthcare professionals in other Indian states as well as LMICs. Through this paper, we hope to contribute to the knowledge on effective capacity-building strategies for public health/health care leaders in resource-constrained settings, in order to strengthen health systems.

## Pedagogical framework, principles, and standards

The HLEP was designed through a collaborative, evidence-based approach for senior health professionals in Gujarat. The training content and design were developed by integrating input from senior government stakeholders, leadership experts, and the participants. An advisory panel of five leadership experts from premier Indian institutions guided leadership competency mapping and curriculum development, aligning it with the government public health, and healthcare sectors, the program design and implementation team consists of members from leadership, social science, and public health domains with experience spanning 18 to 30 years, facilitating applicability and acceptability of the program. Backed by a robust training needs assessment, the initial months were spent in adapting the LEADS competency list to our context through a mix of qualitative and quantitative methodology described elsewhere (16).

The decision to choose the LEADS framework over other leadership competency models was made after reviewing a few other frameworks. We found the Medical Leadership Competency Framework (MLCF) developed by the NHS in United Kingdom was limited to guide doctors in clinical leadership roles (17), whereas we wanted a framework with a wider application for both public health and hospital leaders. The American College of Healthcare Executives (ACHE) Competency Assessment Tool is predominantly management-oriented, viewing leadership as just one of several domains. The Leaders for European Public Health (LEPHIE) framework is more suitable for developing long-term leadership curricula across diverse European health systems (18).

We selected the LEADS framework because of its conceptual breadth, methodological rigor, and adaptability to India’s public health context. Developed in Canada through the Leadership Competencies for Public Health Practice project, it was specifically designed for interdisciplinary public health practice, covering seven disciplines, including medicine, nursing, epidemiology, and health promotion (15, 19). Its structure includes 49 competencies across five domains: Leading Self, Engaging Others, Achieving Results, Developing Coalitions, and Systems Transformation. It offers a validated and internationally recognized foundation, a comprehensive yet straightforward model that addresses both individual and systems-level leadership functions, which facilitates adaptation in LMIC contexts (16). Moreover, the LEADS framework has been successfully applied in various contexts, demonstrating its utility in developing leadership skills to enhance organizational performance (55). The adapted model of the LEADS framework informed the design, delivery, and evaluation of the HLEP.

### Learning environment, learning objectives and pedagogical format

The program was held in an academic setting to create a reflective learning environment and reorient health professionals toward structured learning. The program aimed to foster a comprehensive leadership enhancement, structured around five core domains: leading oneself, engaging others, accomplishing results, building coalitions, and system transformation.

The objectives were to (1) Enhance theoretical understanding and practical proficiency for each of LEADS competency domains, (2) Enable learners to internalize leadership concepts to reflect in behavioral change (3) Enable learners to translate these individual advancements into tangible improvements in health system performance.

**Figure 1:**
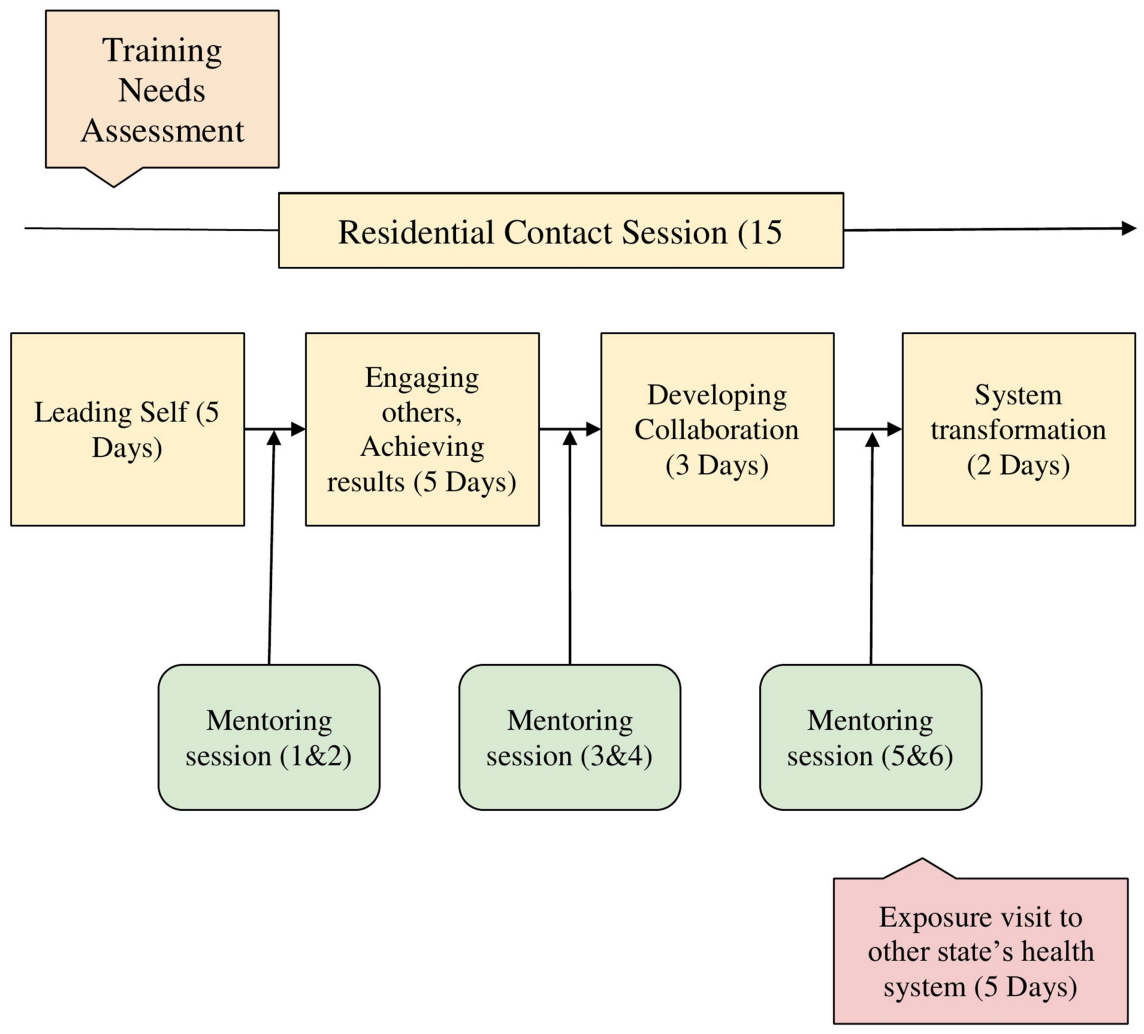
Healthcare leadership enhancement program (HLEP) design. Source: Developed by authors.

The pedagogical format for HLEP incorporates both theoretical knowledge and practical applications, spanning approximately 10 months. It is delivered through (1) four residential sessions (Residency) totaling 15 days (120 h), scheduled at gaps of around 3 months. (2) Six intervening virtual mentoring sessions by experienced practitioners, a total of 132 h, and (3) an exposure visit to other state’s health systems as shown in Figure 1.

Recognizing that adult learners require practical, problem-centered interventions, we anchored the program in Knowles’ Andragogy principles (20, 21), using real-world simulations, peer reflections, and self-directed modules customized to officers’ roles. The pedagogy ensured that theoretical frameworks are translated into actionable competencies, such as Situated Learning (contextualized skill-building through specially developed case-studies and simulations), and Deliberate Practice (repetitive drills on high-impact tasks through role-plays, self-practices).

1 Residencies: Residencies were designed to address the five domains of the LEADS framework. The key content areas are summarized in Annexure 1, and sample sessions from one residency are presented in Annexure 2.

The residency sessions were augmented by various pedagogical tools (see Annexure 3), including case studies, simulations, role-plays, and psychometric assessments that integrate theoretical concepts, contextualizing them to real-world challenges. The program’s unique feature was the two case studies specifically developed for the public health and healthcare, reflecting real-life situations and challenges participants face in their leadership roles. These were developed based on interviews conducted during the initial period of training needs assessment. These cases helped resource persons from diverse fields contextualize their teaching content to the public health and healthcare domain.

2 Mentoring session: We leveraged the expertise of 10 senior officers from the government health department, holding 15–20 years of leadership experience in public health and healthcare. Mentors were briefed about program content and their role in the mentoring process. Each mentor was responsible for guiding four to six participants through bi-weekly virtual interactions scheduled after each residency. Practice assignments assigned at the end of every residency guided participant leaders to apply LEADS learnings into their workplace situations, and mentors to discuss these concepts during mentoring sessions.

Through their practical administrative experience, the mentors facilitated the contextualization of theoretical frameworks, helping participants learn problem-solving through practice assignment exercises designed to address real-world work difficulties. Drawing on their tacit knowledge, the mentors could demonstrate adaptable leadership techniques tailored to real public health and health care environments. The project team members participated in each mentoring session to ensure discussions aligned with the program’s needs and practice assignment queries. Notes were taken during mentoring interactions.

3 Exposure visit: To: (a) facilitate cross-state learning of innovative health system practices; (b) observe leadership-in-action in a new setting; and (c) assess transferable strategies for participants’ home state contexts, the program included a structured one-week exposure visit. In order to optimize the conversion of observed best practices into practical leadership strategies, this immersive component integrated facility visits, debriefing sessions with host leaders, and reflection exercises.
4 Practice Assignments: Post-residency practice assignments required participants to apply learned theories to their workplace situations. For example, participants were required to conduct team analyses using Situational Leadership Theory (22, 23), mapping team members’ competence, motivation, and teamwork levels. They were encouraged to align their leadership style with each team member’s developmental level. They received mentor feedback on adapting approaches to organizational constraints. They were supposed to share their learning and experience of implementing the assignment during the next residency and also during the mentoring sessions. Hence, all the components of the HLEP program converged for a better learning outcome over the period of around 10 months.

### Participants

A total of 150 officers attended the program between August 2022 and March 2025. divided as three cycles. Each cycle consisted of two batches of 25 officers, totaling six batches. To select participants, the Government invited nominations from all 33 districts in Gujarat. Participants were selected based on predefined criteria: a minimum of 15 years of experience, not more than 50 years of age, and a proven track record of efficient work in public health, medical services, or medical education at the state or district levels. Two of the officers dropped out of the program due to personal reasons, resulting in 148 officers. Table 1 indicates the profile of the participants who benefited from the program. After completing training for six batches, the program’s effectiveness was evaluated.

**Table 1:** Participants profile.

Characteristics	Categories	N (148)	%age
Job Role	Public Health	88	59
	Medical Services	39	27
	Medical Education	21	14
Gender	Male	120	81
	Female	28	19
Seniority	Class I (Senior cadre)	71	48
	Class II (Middle Cadre)	77	52
Geographical location of public health doctors	State HQ	6	7
	District HQ	58	66
	Block HQ	17	20
	Primary Healthcare	7	8
Work Experience	Early level (0–10 years)	19	13
	Mid level (11–20 years)	68	46
	Senior level (21–30 + years)	61	41

### Program evaluation

We evaluated the program’s effectiveness through the four levels of the Kirkpatrick framework.: Reaction, Learning, Behavior, and Results. Level 1 is Reaction (the degree to which participants find the training favorable, engaging, and relevant to their work), Level 2 is Learning (estimate participants’ knowledge, confidence, and commitment based on their participation in the training), Level 3 is Behavior (application of learning’s back in their job), Level 4 is Results (leadership role outcomes which occur as a result of the program) (24).

Table 2 outlines the different data collection methods employed at various time points to assess the program’s effectiveness across all four levels comprehensively. We collected participants’ Reactions on a 5-point Likert scale through four feedback forms distributed at various stages of the program, as shown in Table 2. The inputs in all four forms were represented numerically in a table to illustrate participants’ satisfaction with different program components; some of the inputs were taken descriptively too. Quantitative data from feedback forms were analyzed using descriptive statistics to summarize participants’ reactions and overall satisfaction.

**Table 2:** Data source for measuring program effectiveness.

Data source and time point of collection	Content	Levels as per the Kirkpatrick model
Daily feedback form	Captured immediate reactions to each session	Level 1-Reaction
Overall residency feedback forms (administered at the end of each residency)	Measured satisfaction with program’s content, perceived ability to implement learnings, training delivery by resource persons, clearing doubts and ease in approaching, and expertise of resource persons	Level 1-Reaction
Overall program feedback form (administered after the program)	Measured Training transferability, pedagogical tools used, practice assignments (applicability of assignments to their job and better understanding of sessions), and mentoring interactions feedback (usefulness in overcoming practical issues, adequacy of mentoring sessions).	Level 1-Reaction
	Response to the following questions in the Overall Program Feedback form: (a) List out three things you found most insightful in HLEP, (b) Share at least one thing you think will remain with you for the long term due to attending this program.	Level 2-Learning
	Self-reported instances of perceived behavioral change	Level 3-Behavior
The Exposure visit to other state feedback form	Captured information on (a) Facilities selected for visit, transportation—intrastate and interstate, and learnings from the visit, (b) specific best practices participants planned to replicate/adapt. This post-visit structured reflection mechanism collected the input from each participant on what they would like to replicate in their own facility and what they will change as a result of witnessing best practices in another state.	Level 1-Reaction, Level 2-Learning
Pre- and post-training test scores	20-question pre-test based on the five domains of the LEADS framework, covering key leadership competencies addressed in the program. Reflective and scenario-based questions were included to assess conceptual understanding and practical application. The same instrument was administered post-training to measure shifts in knowledge, skills, and self-perceived leadership abilities.	Level 2-Learning
Mentoring interaction notes	Taken during each of the mentoring interactions	Level 3-Behavior
Practice assignments-six practice assignments submitted by the participants (two after 1^st^, two after 2^nd,^ and two after 3^rd^ residency)	Qualitative narratives from the practice assignment	Level 3-Behavior
	Self-reported final work outcomes	Level 4- Results

We assessed learning improvement by comparing pre- and post-training test scores and overall program feedback. For Level three- Behavior, qualitative narratives from the practice assignment were analyzed to extract examples of changed behaviors in participants’ current job settings. Level four- Results were primarily captured through self-reported final outcomes at the workplaces from the practice assignments. Participants were expected to carry out an individual leadership-level collaborative intervention as part of the third practice assignment. Reports on the procedure and results of their work were requested. We organized these qualitative narratives to highlight the program’s overall impact and effectiveness at the individual, team, and organizational levels.

Data for the qualitative analysis were drawn from open-ended feedback forms, mentoring notes, practice assignment submissions, and self-reported work outcomes. All of this textual data was collated into Word files.

An *a priori* coding scheme, derived from session titles and subtitles developed in response to the Training Needs Assessment (16), guided the analysis. Resource persons addressed competencies such as Empathy, Emotional Intelligence, Delegation, Motivation, Communication Skills, Networking, and many others during the four residential contact sessions.

Textual data were charted in five Excel data sheets, one for each of the LEADS dimensions, with further categorization under related titles and sub-titles. The team collectively compared their interpretations and resolved discrepancies through consensus discussions. We then mapped the data and illustrative quotes onto the broader LEADS domains and further synthesized into practical higher-order categories of Individual, Team, and System level outcomes.

## Findings

We present our findings along the four levels of the Kirkpatrick model. First, we examine participants’ immediate reactions to various program components. Next, we explore the extent of learning achieved through the program. The third section assesses behavioral changes in the participants, and finally, the fourth level highlights tangible results derived from the interventions implemented as part of the practice assignments.

### Level 1: reaction

**Figure 2:**
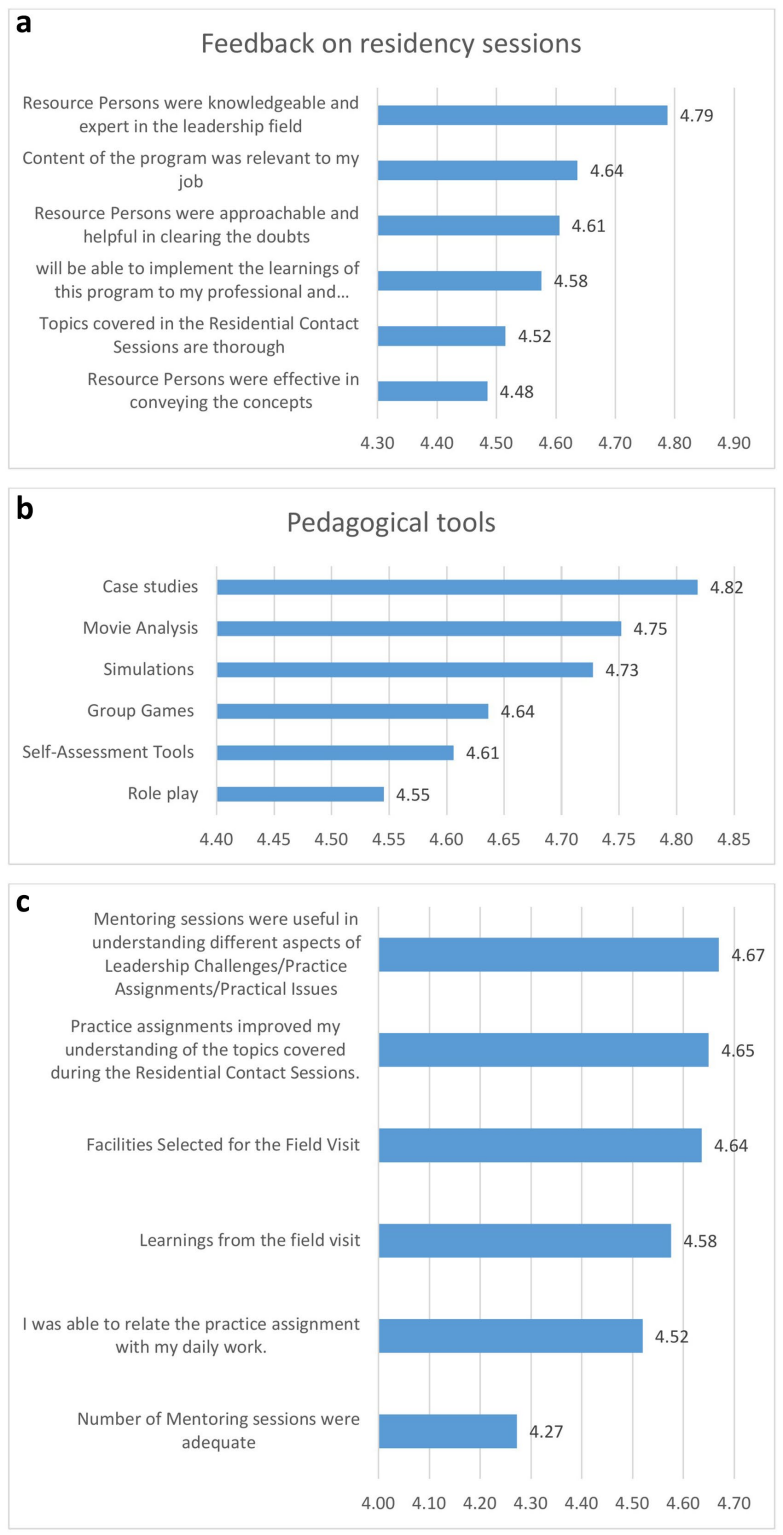
Feedback from the participants. **(a)** Feedback from participant’s on residency sessions; **(b)** Feedback from participant’s on Pedagogical tools; **(c)** Feedback from participant’s on mentoring sessions and practice assignments.

The immediate impressions of participants after each residency were scored on a 5-point Likert scale. The participants’ ratings ranged from 4.48 to 4.79 out of 5. The maximum positive response (Figure 2a) was given to resource person’s knowledge and expertise, followed by their satisfaction with the relevance of the content to their jobs, indicating its alignment with essential job-related skills. However, not all participants felt that the resource person was effective in conveying leadership concepts or in covering the topics comprehensively.

Participants appreciated all pedagogical methods used during training, with the rating ranging from 4.55 to 4.82 (out of 5). Highest rating was given to case studies, followed by movie analysis, and simulations (Figure 2b). There remains room for improvement in using group games, role-play, and self-assessment tools as pedagogical methods.

Among components for feedback about activities to reinforce and apply theory in the classroom, the participants rated practice assignments and mentoring sessions slightly less favorably compared to pedagogical methods used during residencies (Figure 2c). The ratings ranged from 4.27 to 4.67 out of 5. The frequency of mentoring sessions, in particular, received a lower satisfaction score (4.27), suggesting a need to reduce the number of interactions. Participants expressed satisfaction with the quality of mentoring and practice assignments, the selection of field visit sites during the exposure visit. They appreciated the learning opportunities, especially those perceived as transferable to their home states. Their responses highlighted the value of field visits in supporting the program’s objectives. Regarding the overall transferability of the program, the average rating was 4.72 out of 5, indicating high satisfaction with some room for improvement.

The participants’ responses to the open-ended questions were in congruence with the quantitative responses. Some examples are given;

*“The case looks like our (own) story …. you know…. we got to know how to manage the situation when media comes (confronted with the media),”*

*“Simulation was so interesting, I tried it for the first time in life…. I had never thought, games can teach me how to use team skills to reach a goal.”*

*“Now onwards, I am going to watch movies from management lens too, it was very interesting!!”*

Some participants expressed satisfaction with topics such as self-awareness, understanding situational leadership approaches, and structured problem-solving approaches.

### Level 2: learning

Pre- and post-training test scores were measured by administering the 20-question test. Overall, test scores among program participants improved by 24.19% after the training (*p* = 0.002). (Table 3) The paired t-test (*p* < 0.05) indicates that the program had a statistically significant positive effect on their knowledge level and overall learning of leadership competencies.

**Table 3:** Batch-wise pre- and post-training scores.

Batch	Average pre-test score	Average post-test score	% Change
Batch 1	67	87	22.99
Batch 2	72	89	19.10
Batch 3	68	92	26.09
Batch 4	70	93	24.73
Batch 5	65	89	26.97
Batch 6	68	91	25.27
Average % increase in learning			24.19

Apart from these scores, data for “Learning” were captured from the participants’ written open-ended responses in overall program assessment form (117 responses from *N* = 63) to the question—“Most insightful concepts they learnt from the program?” Their enhanced theoretical understanding and practical proficiency for key LEADS competencies were reflected in their self-reports. Most of their responses could be categorized into “self-awareness” and “people management” where they expressed that they learnt the most. Overall, the participants expressed better understanding of their own personalities, ego states, and self-biases. They became more receptive to feedback, improved their self-assessment skills, and learned to manage their ego states, enhancing their ability to navigate inter-personal challenges.

Several participants acknowledged a better understanding about “knowing self”;

*“I never did any personality assessment before, now I know my strength and weakness, it was eye-opening …,”*

*“I learnt about my preferred transactional pattern,”*

*“I never assumed that my fixed-mindset was hindering (my interactions) so much.”*

*“I got to know my personal biases in decision making and judging others. I will be more careful now.”*

Most participants learnt better team management (Engaging others), and dealing with other stakeholders in the system (Developing coalitions),

*“…. I was not using an effective leadership style with my team, now I learnt about the situational leadership concept,”*

*“I understood, which conflict management strategy to use when we get irrational demands from the local Sarpanch (Elected Village leader)” and.*

*“I always used to get anxious at the time of signing a file related to finance or purchase matters, but now I know what to check before signing.”*

Learnings from the exposure visit led to potential Systems adaptations, albeit small; the participants shared;

*“We learnt how effectively they had installed an immunization selfie point and a mirror to check diabetic foot to make the center attractive, we can also do this!”,*

*“We can also have a separate entry in our casualty department for heart-related emergencies……,”*

The participants understood the strategies for managing media and press during crises and demanded more sessions on navigating political interference in their daily roles as district leaders.

### Level 3: behavior

Our participants reported behavioral changes which indicate the internalization of some key leadership concepts.

Realizing that he had been mostly directive with his team, a municipal corporation official shared that “*I never used to think….whether the work (allocated) matches their ability. Now…I try to match the task to their strength and interest, if possible.”*

A frequent self-observation we noticed in the final program feedback was that many participants began to delegate tasks, something they previously hesitated to do. As one participant described, “*My staff appreciated me after seeing changes in my leadership approach, which earlier was more directive. For example, in the execution of vaccine drive, now I am more confident to delegate tasks to them … they are also happy about it*.”

Some participants shared their experiments with the application of ‘negotiation techniques’, discussed during the second residency, such as collaboration between grassroots health workers (ASHAs-Accredited Social Health Activists and SHGs-Self-Help Groups- from rural communities). As shared by them, *“we tried to integrate…….the ASHAs and women of the SHGs, to work together for creating awareness during school health programs at the community level. Both the groups got role clarity and agreed to the common mission, but later it resulted in insecurities.”* This partially successful attempt gave opportunities to understand how collaboration can work and what could be the challenges to be resolved.

While a few reported partial success, many acknowledged positive shifts in their team approaches, which led to improvements in workplace relationships and team functioning. For instance, one Taluka-level health officer shared during the mentoring, “………*I always discuss target achievement with my team. I thought* (*Instead), Let me ask them about their family, their hobbies, and their favorite foods. To my surprise, they started sharing new ideas more openly with me. I could build rapport with them!”* They seemed pleased with their new approach and the associated benefits. The gap between leaders and their teams narrowed, fostering a more participative environment where individuals felt valued and contributed more actively.

Overall, the first and second residencies appeared instrumental in helping participants transition from a directive to a more situational leadership style. They developed self-awareness, strengthened communication skills, and practiced delegation more effectively, enhancing team dynamics and earning appreciation from their subordinates.

After the third residency on “Developing Collaboration,” participants reported a shift in their mindset regarding interdepartmental cooperation. One dental surgeon realized that ‘*one tends to assume that collaboration with others is difficult, but it may not be the case’.* He proactively approached the department head of the tuberculosis program, for collaboration with the public health department, which was successfully accomplished.

However, no behavioral changes could be documented for the final residency dealing with “systems transformation” as there was limited opportunity to collect follow-up data, and participants indicated that system transformation was outside their scope, given the constraints of their roles and hierarchical structures of the health system.

### Level 4: results

From the participants’ reports of the practice assignment in which they designed a targeted intervention for a current leadership issue at work, we could gage participants’ individual advancements being translated into tangible improvements in health system performance. We categorized the responses under the system, team, and individual-level improvements (Table 4).

**Table 4:** Participants’ reported outcomes of practice assignments.

Category	Themes	Quotes
System-level outcomes	External collaboration	“I used concepts of task delegations, interpersonal collaboration and communication to bring awareness and strong linkages between the private and the government system. This resulted in an increase of 22% in TB case notifications by private practitioners and laboratories”—Block Program officer
	Decentralization and communication	“There has been a surge from 12 to 70% in generation of cards for Ayushman Bharat Health Account (ABHA), I carried out multiple meetings with block level officers, identified champions among them who had over-achieved their given targets to set examples for rest to follow, …. with rigorous follow up and tracking down performance till the level of ASHAs, we could achieve this!”—Chief District Health Officer
	Delegation	“I have given my team freedom to re-design the process of beneficiary registration…now with improved process-flow, we could cover more in less time. I used a delegating style for my team members, while simultaneously, showing trust by giving them space to re-design their work for the PMJAY (Pradhan Mantri Jan Arogya Yojana) card enrollment campaign, resulting in the generation of 4,623 new cards. This exceeded daily performance expectations.”—ADHO (Additional District Health Officer)
	Situational leadership approach	“The family planning program achieved 83% of its target by the end of July 2023, reflecting a 27% improvement compared to the previous year. Program implementation activities increased, and inter-sectoral collaboration strengthened. I became more flexible in how to deal with each of my team members as per their level”—Block program officer. One more health officer reported, “Earlier I was very directive but now I am trying to coach my team and sometimes delegate too, hence, the acceptance of Medroxyprogesterone acetate (MPA) injections in my Taluka climbed from 10.48% in July 2022 to 29.31% in July 2023.”
Team-level outcomes	Communication and integration	One hospital administrator shared “Earlier they all used to work in silos, then I started putting them together for tasks requiring support from others…and in no time, this dependency brought positive change!”
	Motivation	I did few team-building exercises with my team to motivate, and it really worked; they became slightly more open to each other.”—Chief District Health officer (CDHO)
	Empowerment	“I gave my counselors an extra responsibility to orient the beneficiary about the program, while taking their history during OPD consultation. They felt empowered, and worked with the medical officer. Their teamwork is really good. I became free to accommodate more patients”—Psychiatrist Mental Health Program
Individual level outcomes	Empathy	“Now I try to be more empathetic while dealing with students with low scores and other behavioral issues, not being impatient as earlier”—Medical College Professor
	Self-realization	“I can devote time to designing new modules as I started delegating small tasks and learnt to put trust in staff, I never realized this earlier.”—State-level training officer.
	Emotional intelligence	“I have got so much control over my anger now…. I remain in my adult ego state whenever dealing with staff negligence or their interpersonal conflict issues.”—District Hospital superintendent.
	Managing stress	“I could practice mindfulness to cope with stress level at my workplace, although I would request to add more sessions on it.”—Medical Officer of Health, Municipal corporation.

The interventions took many different forms; some focused on team-level enhancements, others on self-reflection that resulted in personal transformation, and a number of them dealt with interpersonal disputes within their teams. Some participants used the program’s learning to enhance departmental processes, while others used the residency’s communication techniques to engage with the media and press in productive ways. These varied, small-scale interventions continuously produced favorable results, indicating the HLEP’s usefulness.

In a few team-level interventions, participants facilitated team discussions which later resulted in the formation of closely knit groups with shared interests, where individuals freely expressed new ideas. Participants noted that providing financial and technical support significantly enhanced team performance. It granted them greater autonomy in implementing program activities, and teams made decisions collaboratively. At the individual level, change primarily manifested as behavioral adjustments and a shift in leadership style. Several participants chose to focus on personal interventions, such as managing anger, practicing mindfulness, and reflecting on their leadership approach. While participants did not report major system-level interventions—such as changes in policies, rules, or procedures—they did initiate minor system-level improvements within their sphere of influence, which yielded positive outcomes.

## Discussion

This paper had twofold objectives: to describe the process of developing and implementing HLEP and to present preliminary findings of the evaluation of the program through the Kirkpatrick model.

The development of the HLEP was founded on situational, cultural, context-specific adaptation of the LEADS framework, addressing unique challenges in the Indian healthcare system (16). Despite challenges such as resource constraints, a multi-tiered structure, higher disease burden and the absence of formal leadership training for health professionals (25, 26), our participants were able to apply some of the step-wise learnings from the LEADS competencies to their work settings and experience modest successes.

Designing HLEP around the LEADS framework is a strength of the program, as it ensures a strong theoretical grounding, and a validated comprehensive content. The process of adapting and contextualizing the framework through rigorous TNA (Training Need Assessment), and validation by Indian experts, added to its strength. Yet another strength added by the rigorous TNA was the context-specific teaching material developed to enrich classroom teaching. The HLEP’s intense design spread over 10 months, with almost 11 contact points with participants in the form of residencies, mentoring sessions, and exposure visits, ensured the participants’ continued engagement, as opposed to a one-time leadership training. Such a ‘multiple delivery method’ has proven very effective in numerous other settings too (27).

Aligned closely with the higher levels of the Kirkpatrick Model, particularly in the domains of behavior and results (28), three key components emerged as significant for the effectiveness of the HLEP; (1) the use of context-specific case studies, (2) Practice-based Assignments, and (3) structured mentoring.

Case-based learning promotes critical thinking, contextual analysis, and decision-making skills (29–31). Embedded in real work context, the cases developed for HLEP, bridged the gap between theoretical knowledge with practical application. Most participants found them relatable and relevant to their work contexts.

To facilitate experiential learning and improve retention and application of leadership concepts, the Practice Assignments guided participants to create tools and strategies to solve real-time work challenges (32, 33). However, it was a challenge to customize practice assignments for the variety of finer job roles of individuals. A few participants struggled to fully relate and apply the assignments to their job roles, resulting in some low ratings. A “one-size-fits-all” design in leadership development often fails to account for varying professional roles and organizational contexts (34), and this needs to be addressed through the creation of a larger variety of Practice Assignments.

Mentoring enhances both learning outcomes and leadership behavior by providing individualized guidance, accountability, and reinforcement of concepts (35, 36). Mentoring in between residencies was a strength of the program, as it gave exposure to the participants to a different group of practice leaders and kept up the momentum of learning. The sessions created a safe space for problem-solving, and feedback. However, it was a challenge for some mentors who were new to leadership concepts, to fully internalize the competencies, which the participants had imbibed, and help them to apply the same in their practice assignments. The briefing sessions on LEADS competencies given to the mentors need further augmentation. Though the mentoring sessions were virtual, at times, the participants found it difficult to take time out from their full work schedules. Effective mentoring requires both significant time investment and adequate preparation (35, 36). Participant feedback also reflected these concerns with a comparatively lower rating.

Learning primarily occurred in the domains of “knowing self” in terms of recognizing their personality, leadership styles, and biases, applying leadership concepts to improve self-awareness, interpersonal effectiveness, and decision-making as also shown by Day et al. (56); McCauley et al. (57).

At the team level, “engaging others,” the effects were observable but comparatively moderate. Participants could apply situational leadership approaches, motivational frameworks, and conflict-resolution strategies during their interaction with teams. Several reported improved team coordination, enhanced motivation, and better communication as outcomes of their interventions, indicating a beginning of transfer of learning into team dynamics. Leadership shifts were evident, with participants adopting more supportive, coaching, and delegating styles.

System-level changes, however, were the least visible probably due to external factors that influence training transferability, such as organizational structure, leadership support, opportunities for application, and resource availability. Health leaders working within rigid, target-driven, and hierarchical government systems face structural barriers when attempting to apply newly acquired leadership skills. This echo finding reported in the literature of public sector leadership, observing that leaders in hierarchical systems often face restricted autonomy and policy inflexibility, limiting their ability to drive transformation (37–40). However, the participants reported improved target achievement and increased uptake of programs, particularly in the implementation of some national health programs.

To summarize, while individual and team-level improvements are more readily achievable, system-level changes require enabling policy environments and sustained institutional support, underscoring the complex interplay between individual capability and organizational context in leadership development outcomes (41, 42).

The pedagogical design of HLEP, grounded in the LEADS framework, demonstrates strong potential for adaptation across diverse contexts, including nursing leadership, heads of departments in medical colleges, hospital administrators, and private health systems, where contextual challenges may differ. Its flexibility to address varied organizational and geographic settings enhances its scalability and reach. Future research could explore the effectiveness of fully digital or hybrid delivery models, the role of interprofessional learning in fostering collaboration, and the program’s impact on learner performance and organizational outcomes. While HLEP has been delivered in a hybrid format, complete in-person implementation may be preferable when operational constraints permit. Conversely, fully online delivery may require careful redesign of its practice-oriented, experiential pedagogical tools to preserve engagement and skill application.

Our findings should be interpreted in light of certain limitations. As participants were both self-selected and screened by the government, there is a potential for self-selection bias or selection bias introduced by the screening process. We had established eligibility criteria for participation at the time of roll-out to help mitigate this limitation.

Further, the sample size was limited to 148 senior health officers from Gujarat, which restricts the generalizability of the outcomes to other groups, such as junior officers or professionals from different states and professions requiring leadership. Another limitation was the context-bound nature of the study, leading to findings linked to Gujarat’s specific institutional and cultural environment, making it difficult to apply results uniformly in other settings.

Additionally, the short-term evaluation only captured immediate outcomes at the conclusion of the program, without assessing the longer-term impact on leadership practices or health system performance; longitudinal follow-up is needed for sustainability insights. Lastly, the potential for researcher bias exists, as those involved in program implementation may unintentionally influence the interpretation of data. Measures such as anonymous feedback and future external evaluation can mitigate this risk, but it cannot be entirely eliminated.

## Implications

Evidence suggests that training a critical mass of health professionals is fundamental to embedding and sustaining systemic change (43). Policy interventions, therefore, should aim to scale up leadership development initiatives to sustain health system reforms. Studies highlight that building the capacity of entire teams—rather than focusing on a few individuals—fosters a shared mental model, enhances collective decision-making, and creates a common language among members, all of which are critical for initiating and sustaining change (44).

Institutional and organizational support is essential for optimal transfer of training. This includes cultivating a culture of psychological safety that encourages reflective practice, continuous improvement, and tolerance for failure without fear of negative consequences (27, 45). Complementing this environment, the use of experiential pedagogical tools in the leadership program—such as case studies, simulations, and role-play exercises that mirror real-world challenges—can deepen skill internalization and facilitate the practical application of leadership competencies in practice.

Embedding values that underpin effective leadership within institutions is a long-term and two-way process between leaders and the institutions they serve. Leaders shape and embed organizational culture through their behaviors, and the policies and practices they reinforce (46). Sustaining such values requires alignment of organizational systems and practices with the desired culture (47). In the public sector, embedding democratic values and ethics in leadership is central to building institutions that serve the public interest (48). Institutionalizing such leadership values would be a long-term process that demands rigor, sustained commitment, and the active engagement of both internal leaders and external stakeholders across the wider communities, political leadership, and public systems.

## Data Availability

The raw data supporting the conclusions of this article will be made available by the authors, without undue reservation.
