# Supplementary material for: Enhancing healthcare leadership in Gujarat, India: an effectiveness study
Source: Front Public Health. 2025 Oct 20;13:1677824. doi: 10.3389/fpubh.2025.1677824 (PMC12580349; doi:10.3389/fpubh.2025.1677824)
Supplement: Supplementary file 1 [file Table_1.docx]

**Annexures**

**Annexure 1: Content covered in the program**

| Residency | Theme | Broad content covered |
| --- | --- | --- |
| 1 | Leading self | Self-awareness, emotional intelligence, decision-making, and knowing one's personality, understanding self-constraints and strengths to lead oneself, and mindfulness |
| 2 | Engaging others and Achieving results | Motivating teams, effective communication, achieving team effectiveness, conflict resolution, negotiation, effective delegation, improving team performance and situational leadership |
| 3 | Developing collaborations | Managing media and press, effective networking with external stakeholders, and managing external political dynamics |
| 4 | Systems transformation | Facilitating change, managing transformation in an organization, Design thinking, coping up with resistance to change |

**Annexure 2: Sessions of Residency I (Theme- Leading Self)**

| **Session title** | **Competencies focused** | **Learning Objectives** | **Pedagogy** |
| --- | --- | --- | --- |
| Self-Awareness & Self-Leadership | Enhance self-perception, interpersonal awareness, and self-management | - To identify and understand the self (personality and values) and the importance of self-awareness for leadership enhancement.  - To develop strategies for effective self-leadership and decision-making. | Personality assessments,  Reflective discussion |
| Emotional Intelligence | Enhance empathy & emotional intelligence. | -Understand the concept and importance of emotional intelligence (EQ) in personal and professional success.  -Identify the five core components of emotional intelligence: self-awareness, self-regulation, social skills, empathy, and motivation.  -Develop practical strategies to improve emotional intelligence for better communication, collaboration, and decision-making. | EQ self-test, role-play, class discussion |
| Role Efficacy & Personal Vision | Demonstrate a Personal vision that is clear and convincing | -Assess their current role efficacy and personal vision  -Articulate a personal leadership vision statement that reflects their purpose, values, and aspirations within their professional context | Self-assessment tools, individual presentations |
| Mindfulness | Cultivate constant self-awareness, emotions, habit patterns, and reactions in various situations | -Understand the true meaning of mindfulness and dispel common myths.  -Develop techniques to cultivate presence, awareness, and emotional balance in daily life. | Guided mindfulness practice, and Group exercise to understand components of mindfulness, meditation for breath awareness, and class discussion |
| Power, Influence & Persuasion | Navigating organizational power and politics ethically | - To understand the concepts of power, dependency, and their dynamics within organizations. -  - Differentiate between formal & personal power.  - Understand organizational politics and strategies to manage them.  - Analyze real-world scenarios to understand the consequences of power dynamics and political actions | Case discussions, role-play,  scenario analysis, class discussion |
| Identifying and managing stress | Build resilience & coping mechanisms | -Understand the concept of organizational role stress and its impact on individual and organizational performance.  -Identify the key role stressors that affect employees in various workplace contexts.  -Learn about the stages of stress progression and their psychological and physiological effects.  -Explore effective coping strategies and interventions to manage stress. | Assessment tool measuring role stress, guided relaxation practice, and class discussion |
| Am I ethical | Strengthen ethical decision-making & integrity | -Understand the concept of ethics and its role in personal and professional life.  -Explore the relationship between human values, ethical decision-making, and behavior.  -Learn frameworks for inner governance to build personal accountability and ethical clarity.  -Develop effective communication and relationship habits that support ethical practices.  -Reflect on personal choices and their impact on self, others, and the environment. | Case studies, peer experience, and knowledge sharing, class discussion |
| Transactional Analysis | Improve interpersonal communication | -Understand the concept and origin of Transactional Analysis (TA).  -Identify the three ego states—Parent, Adult, and Child—and their influence on communication.  -Analyze different types of transactions (complementary, crossed, and ulterior) and their impact on relationships.  -Apply TA principles to improve communication and reduce conflicts in personal and professional settings. | Role-play, self-analysis worksheets,  Movie analysis,  Case study analysis, class discussion |
| Decision-Making & Biases | Strengthen evidence-based decision-making | -Understand how perception influences individual and organizational decision-making.  -Identify common perceptual errors and biases that affect judgments.  -Recognize and mitigate decision-making biases to enhance effectiveness | Movie analysis,  Case Study analysis, class discussion |
| Leadership vs. Management in Healthcare | Difference in leadership and managerial competency | - Differentiate between the roles and functions of leadership and management.  -Understand the importance of balancing operational efficiency with visionary transformation.  -Explore key competencies and practices that drive effective leadership in healthcare systems. | Group discussion,  Experience sharing, class discussion |

**Annexure 3: The pedagogical tools used in the program**

| **Method** | **Material and tools** | **Aim / Objective** |
| --- | --- | --- |
| **Case Studies** | Hospital leader’s case,  Public health leader’s case | Understand concepts such as decision-making based on errors and biases, performance appraisal, leadership style, and ego state analysis. |
| **Simulations** | Team building exercises | A team-building exercise involves a group practicing team behaviors by working on a real-life problem just in time to understand how a team works together. |
|  | Simulation on survival | Develop communication, leadership, and negotiation skills within a team. |
|  | Power and influence-based simulation | To Understand the concept of Power and influence |
|  | Reflections on life journey | For reflecting and discovering where participants originate and their life journey |
| **Group Games** | Collaborative puzzle exercise | To introduce the concepts of cooperation and competition and explore positive models for problem-solving |
|  | Mindfulness | To develop greater insight into themselves and the world around them |
| **Movie Analysis** | Movie on decision-making,  Movie on handling a crisis | To implement the learning from the sessions for a better understanding of the concepts of leadership |
| **Self-assessment tools** | Leadership mapping | The purpose is to identify and address the mismatch between what leaders expect and how they behave and to identify the leadership style/behavior used by the participants. |
|  | Personality traits | to understand the familiar personality inherited and implemented by the participants |
|  | Organizational Role Stress (ORS) | To understand the role stress in an organization based on the ten role stressors |
|  | Transactional Analysis | To understand ego states and enable individuals' values, behavior, and thoughts within an organization/team. |
|  | Johari Window | To understand how one receives and gives information about oneself and others |
|  | Psychometric test | The test used for self-awareness during situations |
| **Role Plays** | Practicing communication | To understand and improve interpersonal communication, relationships, and the ability to exchange information within the team |
